# Common genetic etiologies of sensorineural hearing loss in Koreans

**DOI:** 10.1186/s44342-024-00030-3

**Published:** 2024-11-28

**Authors:** Seung Hyun Jang, Kuhn Yoon, Heon Yung Gee

**Affiliations:** 1 Department of Pharmacology, Graduate School of Medical Science, Brain Korea 21 Project, Yonsei University College of Medicine, Seoul, 03722 Republic of Korea; 2 Yonsei University College of Medicine, Seoul, 03722 Republic of Korea; 3 Won-Sang Lee Institute for Hearing Loss, Seoul, 03722 Republic of Korea; 4 Department of Pharmacology, Yonsei University College of Medicine, 50-1 Yonsei-Ro, Seodaemun-Gu, Seoul, 03722 Republic of Korea

**Keywords:** Hearing loss, Genomic landscape, Age-related hearing loss, Genome-wide association study

## Abstract

Hearing loss is the most common sensory disorder. Genetic factors contribute substantially to this condition, although allelic heterogeneity and variable expressivity make a definite molecular diagnosis challenging. To provide a brief overview of the genomic landscape of sensorineural hearing loss in Koreans, this article reviews the genetic etiologies of nonsyndromic hearing loss in Koreans as well as the clinical characteristics, genotype–phenotype correlations, and pathogenesis of hearing loss arising from common variants observed in this population. Furthermore, potential genetic factors associated with age-related hearing loss, identified through genome-wide association studies, are briefly discussed. Understanding these genetic etiologies is crucial for advancing precise molecular diagnoses and developing targeted therapeutic interventions for hearing loss.

## Introduction

The auditory system is a fundamental component of human perception that plays multifaceted and essential roles in daily life. Through the sense of hearing, humans can locate the origins of sounds and differentiate stimuli. The most remarkable aspect of the auditory system, however, is the ability that the human lineage has developed to make sense out of sound. Humans convert sound into highly meaningful representations through language, making the status of hearing unique among the senses [1]. Hearing is essential for communication and normal life in humans.

Hearing loss (HL) is one of the most common sensory disorders, with an incidence of 1 in 500–1000 in newborns imposing a substantial economic burden. Among the various etiologies of HL, genetic factors are responsible for at least half of the congenital cases, and within this subset, more than two-thirds are classified as nonsyndromic hearing loss (NSHL), which refers to isolated hearing loss without the involvement of other organs [2]. To date, more than 150 mutated genes have been identified to cause deafness. Among these genes, 63 are inherited in an autosomal dominant (AD) pattern, whereas 86 are transmitted in an autosomal recessive (AR) inheritance pattern (up-to-date overview of the genetic etiologies of hereditary hearing impairment is available on hereditary hearing loss homepage: https://hereditaryhearingloss.org/).

HL can be classified as pre-lingual or post-lingual, depending on the time of onset. Pre-lingual HL starts before speech development, whereas post-lingual HL emerges after the development of speech. An example of post-lingual HL is age-related hearing loss (ARHL), which affects approximately one-third of adults aged > 65 years [3–5]. Most deafness-causing genes inherited in an AR pattern commonly lead to congenital or pre-lingual HL, whereas those inherited in an AD pattern tend to induce post-lingual HL. Furthermore, the genetic etiologies of pre-lingual and post-lingual HL are considerably different, indicating that clarification of the genomic landscape of both types of HL is clinically important for precise molecular diagnosis.

In this review, we summarize the common genetic etiologies of NSHL in Koreans and the underlying mechanisms contributing to either autosomal recessive or autosomal dominant NSHL. The contribution of genetic factors to age-related hearing loss (ARHL) is also briefly discussed.

## Autosomal recessive NSHL in the Korean population

*GJB2* and *SLC26A4* are the most common causative genes of congenital or pre-lingual HL in East Asian populations [6–9]. Consistent with this, *SLC26A4* was identified as the most prevalent deafness-causing gene, accounting for approximately 20% of prelingual-onset NSHL cases, followed by *GJB2,* in a cohort of Korean patients with hearing loss [10]. Here, we comprehensively reviewed the clinical characteristics of *GJB2* and *SLC26A4*-related hearing loss, in addition to the physiological functions and pathogenesis of HL caused by mutations in these genes.

### *GJB2*

**Fig. 1:**
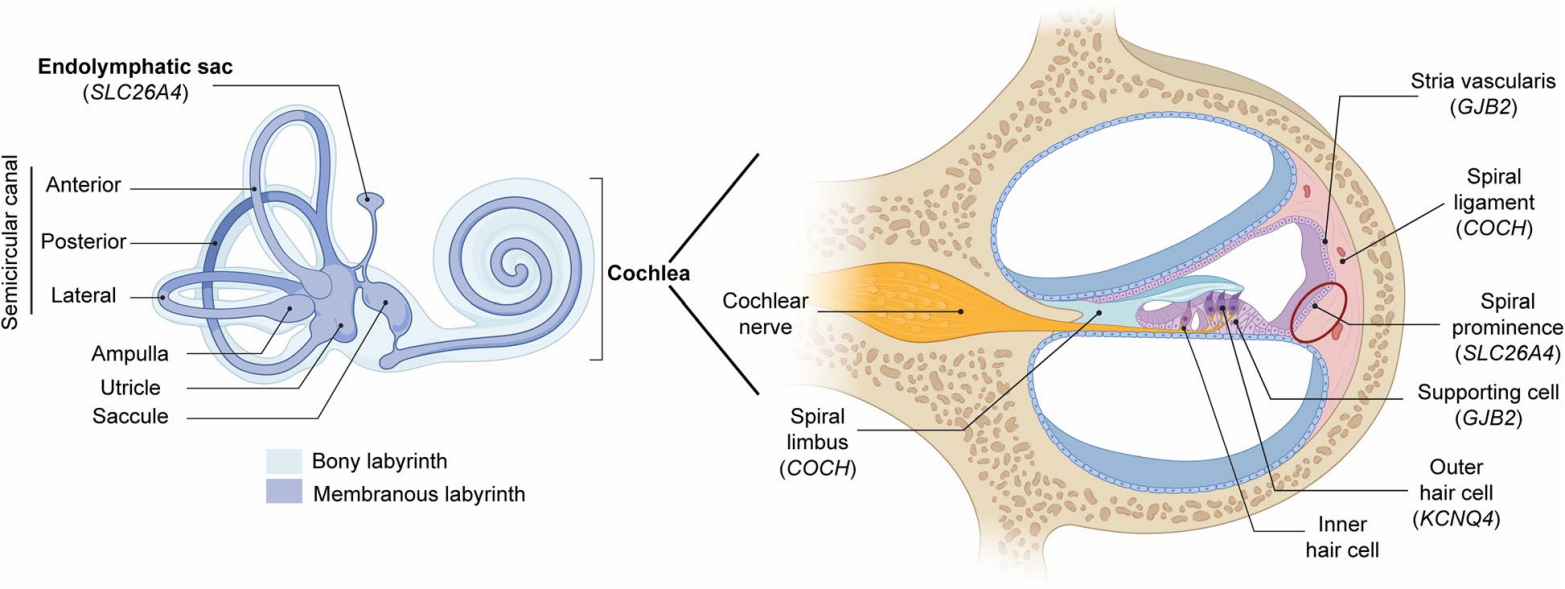
Schematic illustration of inner ear structure. Cell types expressing common deafness-causative genes found in Koreans are indicated

Gap junction protein 26 (connexin 26; CX26), which is expressed in non-sensory cells of the cochlea (Fig. 1), is encoded by the *GJB2* gene [11, 12]. CX26, along with connexin 30 (CX30), is one of the most abundantly expressed gap junction proteins in the inner ear and co-assembles to form heteromeric and heterotypic channels in cochlear gap junction plaques (GJPs) [11, 13, 14]. Most mutations in the *GJB2* gene cause autosomal recessive deafness 1 (DFNB1), a leading form of NSHL [11], whereas a few mutations in the *GJB2* gene have also been reported to cause autosomal dominant deafness 3 (DFNA3) [15, 16]. This review focuses on the phenotypes and pathogenesis of DFNB1.

The degree of hearing loss in DFNB1 patients varies widely, ranging from profound deafness at birth to mild, progressive HL in late childhood [17, 18]. The auditory phenotype is highly dependent on the specific *GJB2* mutation [17, 19]. Patients with bi-allelic truncating mutations typically experience substantially more severe HL than those with bi-allelic nontruncating mutations. Several mutations, including p.M34T, p.V37I, and p.L90P, are associated with mild-to-moderate HL, whereas the c.35delG mutation often results in severe hearing impairment [11, 19, 20].

A study involving the genetic screening of 2072 newborns with normal hearing revealed that the carrier frequency of GJB2 pathogenic mutations (p.V37I, p.G45E, p.R143W, c.176_191del, c.235delC, c.292_298dup, c.299_300delAT, and c.605ins46) was 3%, which corresponds to an allele frequency of 1.49%, in the Korean population [21]. Among the pathogenic variants identified in that study [21], the p.V37I (allele frequency, 0.68%) and c.235delC (0.63%) mutations were the most prevalent (Table 1), which is consistent with their high frequency in East Asians [17]. The p.V37I mutation, the most common *GJB2* mutation in the Korean population, is a missense allele associated with mild HL, which progresses steadily [19–22]. The c.235delC is the second most common mutation, resulting in the premature termination of translation and production of a truncated protein [21, 23]. Although the c.235delC mutation is commonly associated with severe-to-profound HL, the hearing phenotype varies, with a considerable number of patients exhibiting asymmetric HL [23].

**Table 1:** Common genetic variants for autosomal recessive and autosomal dominant sensorineural hearing loss in Koreans

rsID number	Chromosome Position (hg38)	Reference Allele	Alternate Allele	Gene	Coding change	Consequence	Korean AF^a^	Inheritance
rs72474224	chr13:20189473	C	T	*GJB2*	NM_004004.6; c.109G > A; p.Val37Ile	Missense	0.009495	Autosomal recessive (DFNB1A)
rs80338943	chr13:20189346	AG	A	*GJB2*	NM_004004.6; c.235delG; p.Leu79Cysfs*3	Frameshift	0.005600	
rs111033313	chr7:107683453	A	G	*SLC26A4*	NM_000441.2; c.919-2A > G	Splicing acceptor	0.001062	Autosomal recessive (DFNB4)
rs111033220	chr7:107690203	C	T	*SLC26A4*	NM_000441.2; c.1229C > T; p.Thr410Met	Missense	0.000380	
rs121908362	chr7:107710132	A	G	*SLC26A4*	NM_00441.2; c.2168A > G; p.His723Arg	Missense	0.004730	
rs1271250198	chr1:40784233	T	C	*KCNQ4*	NM_004700.4; c.140 T > C; p.Leu47Pro	Missense	0.003860	Autosomal dominant (DFNA2)
-	chr14:30877602	G	A	*COCH*^b^	NM_004086.3; c.113G > A; p.Gly38Asp	Missense	-	Autosomal dominant (DFNA9)
rs924049830	chr14:30878911	C	T	*COCH*^b^	NM_004086.3; c.340C > T; p.Leu114Phe	Missense	-	

^a^KOVA.v2 (Korean Variant Archive for a reference database of genetic variations in the Korean population; https://kobic.re.kr/kova/)

^b^Common variants of *COCH* gene in Koreans have been identified based on reports from multiple independent studies [4, 24–26]

Intracellular gap junction channels (GJCs) formed by CX26 and CX30 are involved in recycling potassium ions (K^+^) and exchanging small molecules, such as glucose, second messengers, adenosine triphosphate (ATP), and miRNAs in the cochlea [11, 13, 14]. GJCs also play a crucial role in energy supply to the cochlear sensory epithelium and inner ear calcium ion signaling, facilitating the diffusion of inositol 1,4,5-triphosphate, a Ca^2+^-mobilizing secondary messenger [11, 27–30]. As the function of GJCs is essential for K^+^ recycling within the inner ear, mutations in the *GJB2* gene result in a drastically reduced GJP area, eventually leading to diminished endocochlear potential within the scala media, which is considered the main pathogenic mechanism underlying *GJB2*-related HL [13, 14, 31–33]. However, recent studies have suggested that impaired K^+^ circulation is not the sole pathological mechanism underlying *GJB2*-related HL [34, 35]. Whereas the mechanism of HL caused by *GJB2* mutations remains unclear, malformation of the organ of Corti is considered a contributing factor to HL [13]. In mouse models with CX26 abnormalities, researchers observed the impaired opening of the tunnel of Corti (TC) and Nuel’s space (NS) [36, 37]. Unopened TCs and an abnormal NS have also been reported in *GJB2* carriers [13]. Given that the opening of the TC and the formation of NS are important for hearing acquisition and that impaired development of the organ of Corti potentially causes severe HL, these observations provide insight into the pathogenesis of *GJB2*-related HL [13, 38].

### *SLC26A4*

The *SLC26A4* gene encodes the pendrin protein, a non-specific anion exchanger [39]. Pendrin is expressed in restricted tissues and is present in the inner ear, kidneys, and thyroid [40]. *SLC26A4* is the causative gene of nonsyndromic autosomal recessive hearing loss, DFNB4, which is associated with an enlarged vestibular aqueduct (EVA) [39]. Mutations in *SLC26A4* occasionally cause Pendred syndrome, a syndromic hearing loss accompanied by goiter and hypothyroidism [41, 42]. Thus, the *SLC26A4* gene serves as a common genetic denominator in both syndromic and nonsyndromic HL. In this review, *SLC26A4*-associated HL refers to DFNB4 and Pendred syndromes.

*SLC26A4*-associated HL manifests as fluctuating and progressive sensorineural hearing loss with a variable range of inner ear malformations [43], such as EVA and incomplete partition type II (IP-II) [39]. EVA is a hallmark feature of *SLC26A4*-associated HL and is observed either unilaterally or bilaterally [39, 43]. Other clinical manifestations include vestibular dysfunction presenting as episodic rotatory vertigo [39, 44].

Although the pathogenic link between endolymphatic sac dysfunction and HL remains unclear, EVA in *SLC26A4*-associated HL is likely attributable to impaired endolymph absorption in the endolymphatic sac during inner ear development [39, 45]. Pendrin is expressed in the apical membrane of the spiral prominence and outer sulcus in the cochlea, transition cells of the vestibular organs, and mitochondria-rich cells (MRCs) of the endolymphatic sac in the murine inner ear (Fig. 1) [39, 46]. MRCs, comprising approximately 30% of endolymphatic epithelial cells, express numerous ion transport genes, including *SLC26A4*. Disruption of the regulation of endolymph absorption during inner ear development via MRCs is considered the underlying mechanism of EVA [45]. Given that *SLC26A4* is mainly expressed in the endolymphatic sac and spiral prominence, but not in the organ of Corti, where mechano-electrical transduction and activation of the cochlear nerve occur, SLC26A4-associated HL may be secondary to the dysfunction of the endolymphatic sac. Dysfunction of the endolymphatic sac results in the perturbation of the homeostatic maintenance of endolymphatic pH and scala media enlargement, which eventually leads to the degeneration of the stria vascularis and hair cells in the organ of Corti [47–49].

Pendrin plays a critical role in the acquisition of normal hearing during certain periods of inner ear development. A murine model showed that pendrin expression was required from embryonic day 16.5 (E16.5) to postnatal day 2 (P2) for the development of normal hearing [50]. This time interval corresponds to the period during which rapid maturation of the inner ear occurs in mice [51, 52]. The precise temporal requirement of pendrin emphasizes that the therapeutic time window must be prudently set for the treatment of *SLC26A4*-associated HL.

In the Korean population, the prevalent *SLC26A4* mutations include p.H723R (allele frequency in Koreans according to KOVA.v2 [53]: 0.473%), c.919-2A > G (0.1062%), and p.T410M (0.0380%) (Table 1) [54]. The missense mutations, p.H723R and p.T410M, exhibit protein-folding defects, resulting in retention within the endoplasmic reticulum (ER), thus inhibiting the protein from reaching the plasma membrane [55, 56]. The c.919-2A > G mutant is a splicing variant that causes skipping of exon 8, resulting in premature termination of translation [57]. Patients with *SLC26A4* mutations often exhibit variable degrees of residual hearing and inner ear malformations depending on the specific mutation they carry. In both p.H723R and p.T410M mutations, a portion of the functional pendrin is expressed in the plasma membrane, with the p.T410M mutation demonstrating a higher surface expression ratio. This explains the better residual hearing experienced with p.T410M mutations than with p.H723R mutations. In c.919-2A > G mutants, the leaky 3′ original splice site allows for the production of normally spliced transcripts, which might be responsible for better residual hearing in affected individuals. The ratio of accompanying inner ear malformations, specifically incomplete partition type II (IP-II), differs across genotypes, with more IP-II observed in p.H723R homozygotes than in other genotypes [54].

## Autosomal dominant NSHL in the Korean population

For HL inherited in an autosomal dominant pattern, genetic etiologies are more heterogeneous than those for autosomal recessive NSHL, and the exact contribution of each gene responsible for autosomal dominant NSHL varies depending on ethnicity. However, among Korean patients with autosomal dominant NSHL, the most prevalent causative genes are *KCNQ4* and *COCH* (Table 1). This review presents the clinical characteristics and physiological functions of *KCNQ4* and *COCH*, and the pathogenic mechanisms of HL caused by these genes.

### *KCNQ4*

KCNQ4 (Kv7.4) is a voltage-gated potassium channel encoded by the *KCNQ4* gene that plays an important role in auditory function [6]. Predominantly localized in the basolateral membrane of outer hair cells (OHCs) (Fig. 1), KCNQ4 causes nonsyndromic autosomal dominant hearing loss (DFNA2) when mutated [58, 59]. In South Korea, approximately 4% of HL cases are caused by mutations in the *KCNQ4* gene [6]. The KCNQ4 channel is involved in the formation of M-type potassium currents that repolarize OHCs, reduce cell excitability, and regulate numerous physiological responses [60–62]. KCNQ4 is essential for recycling potassium ions, maintaining resting membrane potential, and ensuring osmotic equilibrium [60, 63].

The KCNQ4 protein consists of 695 amino acids and contains six transmembrane domains (S1–S6) [64]. Among these are four voltage-sensor domains (S1–S4) and a P-loop region residing between the transmembrane domains S5 and S6, with both N- and C-termini located intracellularly [60, 65]. The majority of DFNA2-causative KCNQ4 variants are clustered in the S5–S6 region, which surrounds the ion-permeating pore region [60]. These variants affect channel activity and disrupt potassium ion recycling in the inner ear [6]. Because KCNQ4 channels form homo- or heteromeric assemblies of four pore-forming subunits, mutations in a single subunit can impair channel function, leading to dominant–negative suppression [60, 66].

The underlying mechanism of DFNA2 involves the progressive degeneration of OHCs due to cellular stress caused by chronic depolarization and the accumulation of intracellular Ca^2+^ [6, 67]. While HL in DFNA2 is initially mild at low frequencies and moderate at high frequencies at younger ages, HL progresses over time, with most individuals developing severe-to-profound high-frequency HL by 70 years of age [66, 68]. This phenotype is recapitulated in various mouse models that exhibit progressive ski-sloping hearing loss with selective degeneration of OHCs, especially in the basal turn [67, 69–71]. Overall, these results suggest a critical functional role of KCNQ4 in the mammalian OHCs to maintain normal hearing.

The pathogenesis of HL caused by mutations in the *KCNQ4* gene varies according to the genotype. Variants, such as p.L274H, p.L281S, p.G296S, and p.G435Afs*61, have been reported to reduce the surface expression of KCNQ4 proteins, leading to the accumulation of mutant proteins in the ER [6, 72]. Impaired trafficking of KCNQ4 due to these variants can be partially recovered by treatment with molecular or chemical chaperones [6, 73]. In contrast, most missense variants, including p.L47P, p.S185W, p.R216H, p.W276S, p.R331Q, p.R331W, p.R447W, p.V672M, and p.S691G, exhibit different pathogenic mechanisms. These mutant proteins reach the plasma membrane normally, but the mutations lead to the impairment of K^+^ current or channel conductance, resulting in a dominant–negative effect on normal channel function [6, 74]. While the channel activity of N-terminal or C-terminal mutant KCNQ4 proteins (such as p.L47P, p.S185W, p.V672M, and p.S691G) can be rescued using KCNQ activators, such as retigabine or zinc pyrithione, pore-region variants, such as p.W276S, p.R331Q, and p.R331W, do not respond to these activators, highlighting the need for different therapeutic approaches based on specific mutations [6, 74, 75].

### *COCH*

DFNA9 is a post-lingual, progressive NSHL caused by mutations in the *COCH* gene, which is frequently mutated in AD NSHL cases [4, 76–78]. Patients with DFNA9 exhibit variable vestibular symptoms, ranging from normal vestibular function to episodic vertigo or persistent imbalance [76, 77, 79, 80]. HL in DFNA9 patients typically begins between 20 and 30 years of age, initially affecting high frequencies and ultimately progressing to severe-to-profound levels across all frequencies by the sixth decade of life [4, 77].

The *COCH* gene encodes cochlin, a major non-collagenous protein in the extracellular matrix of the inner ear (Fig. 1) [4, 80]. Although it is abundantly expressed in the inner ear, eye, and spleen, its functional role is critical for maintaining normal auditory function [80, 81]. Structurally, cochlin consists of an N-terminal Limulus factor C, cochlin, and a late gestation lung protein Lgl1 (LCCL) domain, along with two von Willebrand factor A-like (vWFA) domains [4, 79, 81]. Mutations in the LCCL domain often cause HL accompanied by vestibular symptoms [4, 79, 82]. Conversely, mutations in the vWFA domain predominantly result in HL, without substantial vestibular symptoms. The onset of HL also varies by genotype, with individuals harboring vWFA mutations experiencing earlier onset than those harboring LCCL domain mutations [79]. This suggests differential pathogenesis of HL depending on specific mutations in cochlin.

Cochlin plays an important role in the innate immune response in the inner ear [81]. This response is critical for the maintenance of auditory function by protecting the organ of Corti from bacterial and viral invasion [81, 83]. However, the activation of the innate immune response in the cochlea often induces excessive inflammation, leading to collateral damage to the organs of Corti and eventual hearing deterioration [84]. Therefore, fine regulation and spatiotemporal control of the innate immune response are essential for efficient pathogen elimination and the minimization of post-inflammatory damage in the inner ear. In murine models, cochlin has been shown to perform a protective role during bacterial infection. During bacterial infection, the N-terminal LCCL domain is cleaved and secreted into the scala tympani, where it recruits neutrophils and macrophages and sequesters bacteria, thereby preventing pathogens and immune cells from accessing the organs of Corti [81]. Consequently, HL resulting from mutations in the LCCL domain may be attributed to defects in the innate immune response of the inner ear. However, the exact function of vWFA domain remains unknown. Although cytotoxicity, aggregate formation, and impaired post-translational cleavage are the proposed mechanisms for HL caused by vWFA domain variants, further investigation using in vivo mouse models is required to elucidate the physiological role of the vWFA domain in the inner ear [4].

## Age-related hearing loss

ARHL, also known as presbycusis, is a progressive and irreversible sensorineural form of HL that is caused by aging [5, 85, 86]. According to previous reports, HL is the primary cause of global years lived with disability (YLDs) in individuals older than 70 years, affecting 25–50% of people in their seventies [5, 87–89]. ARHL is also associated with tinnitus, social withdrawal, depression, cognitive decline, and dementia, contributing to an annual global economic burden exceeding $981 billion [89–93]. Despite these substantial impacts, governmental and industrial efforts to address HL are relatively limited compared to other diseases of similar prevalence, and there is currently no available preventive treatment for ARHL [87, 89].

ARHL is a complex condition with considerable variability in onset, severity, and progression among individuals [94]. Unlike early-onset genetic HL, which is often determined by monogenetic factors, the cause of ARHL is heterogeneous and involves both environmental and polygenic factors [89, 91]. Although environmental risk factors for ARHL, such as prolonged exposure to loud occupational noises, are well documented, little is known about genetic factors and the underlying cellular and molecular mechanisms remain unclear [85, 91, 94, 95].

The identification of risk loci for ARHL is imperative in understanding the biological mechanisms by which these variants contribute to HL [96]. Recent genome-wide association studies (GWAS) have identified multiple genetic variants associated with the development of ARHL [87, 89, 94]. Ivarsdottir et al. performed a GWAS meta-analysis of 121,934 ARHL cases and 591,699 controls from two non-overlapping Icelandic datasets and the UK Biobank and identified 51 sequence variants associated with ARHL [94]. More recently, Trpchevska et al. conducted a meta-analysis of 17 independent cohorts comprising 147,997 individuals with clinically diagnosed and self-reported HL and 575,269 controls, and identified 48 important loci [89]. We analyzed the allele frequencies of 13 variants commonly found in both studies within the East Asian and Korean populations (Table 2), highlighting the importance of previously identified loci in the pathogenesis of ARHL in East Asians [89, 94, 96].

**Table 2:** Age-related hearing loss-associated loci commonly identified in recent genome-wide association studies

rsID number	Chromosome position (hg38)	Effect allele	Other allele	Gene	Coding change	Consequence	East Asian AF^a^	Korean AF^b^	Odds ratio
rs36062310	chr22: 50549676	A	G	*KLHDC7B*	NM_138433.5; p.Val504Met	Missense variant	0.00009022	-	1.027
rs10901863	chr10:125123701	T	C	*CTBP2*		Intron variant	0.1782	-	1.011
rs5756795	chr22:37726115	C	T	*TRIOBP*	NM_001039141.3; p.Phe1187Leu	Missense variant	0.5642	0.603120	0.992
rs9493627	chr6:133468590	A	G	*EYA4*	NM_004100.5; p.Gly277Ser	Missense variant	0.3518	0.391820	1.009
rs67307131	chr11:118609508	C	T	*PHLDB1*		Intron variant	0.3099	-	0.992
rs11238325	chr7:50785454	C	T	*GRB10*		Intron variant	0.7107	0.711810	1.007
rs6545432	chr2:54590546	G	A	*SPTBN1*		Intron variant	0.4387	0.467130	1.007
rs7525101	chr1:165139894	T	C	*LMX1A*		Intergenic variant	0.6343	0.597030	1.006
rs143796236	chr17:81528943	T	C	*FSCN2*	NM_001077182.3; p.His138Tyr	Missense variant	0.00002277	-	1.035
rs13171669	chr5:149221680	G	A	*ABLIM3*		Intron variant	0.5945	0.556470	0.942
rs920701	chr13:75842965	C	T	*LMO7*		Intron variant	0.3611	0.354260	0.942
rs11881070	chr19:2389142	T	C	*TMPRSS9*		Upstream gene variant	0.5452	0.563680	0.942
rs143282422	chr10:71617355	A	G	*CDH23*	NM_022124.6; p.Ala366Thr	Missense variant	0.00004456	-	1.032

*AF* allele frequency

^a^gnomAD v4.1.10 browser (https://gnomad.broadinstitute.org/; accessed on 16 August 2024)

^b^KOVA.v2 (Korean Variant Archive for a reference database of genetic variations in the Korean population; https://kobic.re.kr/kova/)

As ARHL is a multifactorial disease, identifying the primary pathogenic mechanisms specifically responsible for ARHL is challenging. Some studies have reported an enrichment of ARHL risk loci in the stria vascularis of the inner ear, as well as an increased unfolded protein response in the stria vascularis, which may contribute to the increased risk of ARHL [85, 89]. A recent study identified hair cells as the primary cell type responsible for ARHL [87]. Therefore, further investigation is required to clarify the pathogenesis of ARHL.

## Conclusion

The genetic basis of NSHL in the Korean population elucidates the key genes responsible for both autosomal recessive and dominant inheritance patterns. Pathogenic mechanisms vary widely, from ion channel dysfunction to impaired protein trafficking, and are mutation-specific. Ongoing GWAS have identified potential genetic risk factors for ARHL, although further research is needed to elucidate the underlying molecular pathways. Understanding these genetic etiologies is crucial for advancing precise molecular diagnoses and developing targeted therapeutic interventions for hearing loss.

## Data Availability

No datasets were generated or analysed during the current study.
